# Orthodontically Induced External Root Resorption: A Finite Element Analysis

**DOI:** 10.3390/jcm15072503

**Published:** 2026-03-25

**Authors:** Radu-Andrei Moga, Cristian Doru Olteanu, Ada Gabriela Delean

**Affiliations:** 1 Department of Odontology, Endodontics and Oral Pathology, School of Dental Medicine, University of Medicine and Pharmacy Iuliu Hatieganu, Str. Motilor 33, 400001 Cluj-Napoca, Romania; ada.delean@umfcluj.ro; 2 Department of Orthodontics, School of Dental Medicine, University of Medicine and Pharmacy Iuliu Hatieganu, Str. Avram Iancu 31, 400083 Cluj-Napoca, Romania

**Keywords:** orthodontically induced external root resorption, intact periodontium, orthodontic movements, finite element analysis, failure criteria

## Abstract

**Background/Objectives**: This finite element analysis (FEA) assessed stress distribution in the tooth and dentin within an intact periodontium under 4 N of force and five orthodontic movements (intrusion, extrusion, rotation, tipping, and translation), using four failure criteria commonly used in numerical dental studies. Secondly, differences between brittle- and ductile-like failure criteria were found, and the most accurate criterion was determined. Additionally, movements more prone to inducing external orthodontic root resorption were assessed. **Methods**: Using nine 3D models of the second lower premolar, 180 numerical simulations were performed. The models were anatomically accurate based on CBCT scans. FEA employed the brittle-like Maximum Principal (MaxP), Minimum Principal (MinP), and ductile-like Von Mises (VM) and Tresca (T). **Results**: The results showed that tipping was less prone to external orthodontic root resorption than translation, extrusion, intrusion, and rotation, which showed areas of high stress concentration in the cervical third of the root. High-stress areas were visible only when the dentin-pulp-NVB components were separately analyzed, and not when the entire tooth structure was assessed. Only by correlating the qualitative with the quantitative results could the difference between brittle-like and ductile-like failure criteria be seen. **Conclusions**: In total, 4 N of applied orthodontic force can induce limited islands of external orthodontic root resorption (intrusion–extrusion on the vestibular side, rotation–translation on the lingual and distal–lingual sides). The ductile-like failure criteria maintained the accuracy of the results across all FEA simulations, while the brittle-like criteria showed various quantitative and qualitative inconsistencies.

## 1. Introduction

Orthodontic movements are among the most common causes of external root resorption [1,2], with reported prevalence ranging from 0.02–2.3% [3], 2–5% [4], and 14.8% [5] to 20–100% [6,7,8]. It usually occurs due to periodontal ligament (PDL) and/or subepithelial cement damage in the cervical third, initially with a reduced depth in dentine, followed by progressive resorption apicocoronally and circumferentially, if the etiologic factors are not identified and eliminated [9]. Orthodontic external root resorption can affect not only the dentine and cementum of the root, but also the crown [6,10,11].

When assessing external root resorption in the cervical third, the differences between orthodontic and occlusal causes (e.g., abfraction) must be acknowledged, as there are fundamental biomechanical, behavioral, and topographical differences. The loss of hard tissue will fundamentally alter the tooth’s biomechanical behavior.

External orthodontic root resorption is a multiple-clinical-phase, unavoidable [5,12], self-limiting, irreversible, and pathological process [3,11]. The severity depends on the amount of applied orthodontic force, the type of movement, the application direction, the local anatomy, and the time [6,10,11,13,14,15,16,17,18,19]. Regarding the optimal amount of force, which is still a subject of controversy, 0.28–3.31 N was reported to be relatively safe [20,21,22]. The pathogenesis recognizes the importance of the PDL’s role in the process, but also the fact that after 15–25 days of force appliance, the destruction is usually irreversible [13,14]. The local ischemic risks prone to induce resorption are related to the exceeding of the maximum hydrostatic pressure (MHP) of 4.7–12.8–16 KPa [8,13,14,15,16,17]. Some of the orthodontic movements are more prone to resorption than the others, with cervical third resorption reported to be induced by extrusion, middle third lacunae induced by rotation, and apical third resorption induced by intrusion [4,8,23,24,25].

To be able to investigate and assess these issues, finite element analysis (FEA) is currently largely used in dental studies [2,26]. There are reports of changes in tissue physical properties from in vitro studies [27], along with design and validation issues [26], thus revealing FEA to be the most accurate method of study [13,14,15,16,17,28,29,30,31].

Few new FEA dental analyses of external orthodontic root resorption are currently available in the research field [13]. There are some new studies investigating the abfraction [3,28] and stress distribution in endodontically treated molars [29] and incisors [32,33] using the Von Mises (VM) criterion under 100–300 N of masticatory forces, displaying extremely extended areas of root cervical third stress, with fundamentally different biomechanical color-coded stress display from orthodontic-induced resorption. Other VM FEAs focused on the general stress distribution induced by the orthodontic aligners, but without focusing on resorptive processes [34,35]. It must be acknowledged that there are fundamental differences in terms of depth and surface extensions as well as topography between abfraction (large, deep, and extended cervical third stress [3,28,29,32,33]) and orthodontically induced resorption (small, more superficial, and displayed as small islands [8,13,24,25,36,37,38,39,40]).

A recent FEA study reported cervical root resorption for 0.6 and 1.2 N using the Tresca (T) criterion, with localized islands of resorption in the dentine–cementum component, and with rotation and translation more prone to resorptive processes [13]. However, no comparative study between the different failure criteria and higher amounts of orthodontic force was found [3,41]. Moreover, there are inconsistencies regarding the failure criteria to be used in dental FEAs to produce accurate results [8,24,25,32,33,36,37]. There are numerous FEA studies that, by not respecting the mandatory boundary conditions established by the engineering field for FEA [13,14,15,16,17], reported results with limited accuracy [20,21,22,24,25,36,37]. There is no clear data on the proper criteria or boundary conditions to be employed.

External root resorption in orthodontics can be unexpectedly present during dental treatment; thus, anticipating biomechanical clinical behavior is important. Such biomechanical studies can be conducted using a routine CBCT scan. With clinical advances in artificial intelligence, such FEAs will soon be fully automated and widely available in everyday clinical practice. This will have a significant impact on the prognosis of clinical treatment and will be extremely beneficial from the patient’s point of view by improving treatment outcomes. Only a few FEA simulations have been applied to this type of resorption [8,24,25,37], and only one was from the past five years [13].

This study aimed to assess the stress distribution patterns in the tooth and its dentine in intact periodontium for 4 N and five orthodontic movements (intrusion, extrusion, rotation, tipping, and translation), and four failure criteria commonly used in dental FEA. A secondary aim was the assessment of the differences between brittle- and ductile-like failure criteria, intended to identify which is/are the most accurate in describing biomechanical behavior. A third aim was to assess which movements are the most prone to induce external orthodontic root resorption.

## 2. Materials and Method

This study is the result of a stepwise research project (nr. 158/02.04.2018) investigating the biomechanical behavior of dental tissues under orthodontic loads [13,14,15,16,17].

The study included nine patients (sample size of nine, mean age of 29.81 ± 1.45 years, 4 males and 5 females). Each patient was radiologically examined using cone-beam computed tomography (CBCT, ProMax 3DS, Planmeca, Helsinki, Finland, voxel size 0.075 mm) to assess treatment needs. From the CBCT images, the second lower premolar area, including the neighboring teeth, was selected. Each of these nine patients had varying degrees of bone loss, up to 2 mm. The inclusion criteria were various levels of periodontal breakdown mainly at the cervical third of the root, non-inflamed periodontium, no mandibular tooth loss, intact teeth, no malposition, an indication for orthodontic treatment, and willingness to be regularly monitored.

Numerical studies (finite element analysis/FEA) can simulate a large and countless number of local conditions; thus, they require only a single sample. Our study used nine 3D models and 180 simulations to enhance the accuracy of the results.

**Figure 1:**
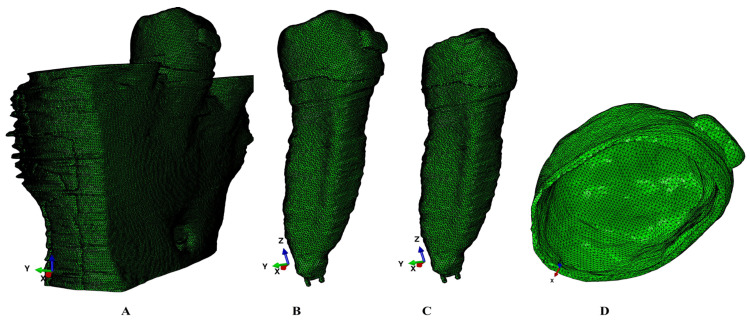
Mesh model of one of the nine 3D models: (**A**)—2nd lower right premolar model intact periodontium; (**B**)—tooth structure with enamel, dentine, dental pulp, NVB, and stainless-steel bracket; (**C**)—dentine structure with dentine, dental pulp, and NVB; (**D**)—enamel component with stainless-steel bracket base.

**Figure 2:**
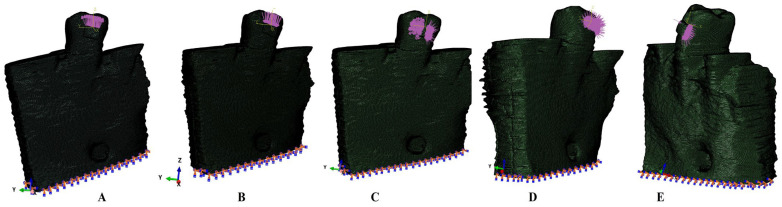
Mesh model of one of the nine 3D models with vectors: (**A**)—extrusion; (**B**)—intrusion; (**C**)—rotation; (**D**)—tipping; (**E**)—translation.

For each CBCT scan, the manual segmentation process was performed using Amira 5.4.0. (Visage Imaging Inc., Andover, MA, USA) to identify each anatomical component (enamel, dentine, cementum, dental pulp, neuro-vascular bundle/NVB, periodontal ligament/PDL, and cortical and trabecular bone) (Figure 1). The PDL had a variable thickness of 0.15–0.22 mm. A clear separation between dentine and cementum was not possible, and since they had similar physical properties (Table 1), they were reconstructed as dentine. The missing bone and PDL were reconstructed to obtain 3D models with intact periodontium (Figure 2). The base of a stainless-steel bracket was reconstructed on the enamel vestibular side. The 3D models included only the second lower premolar, while the alveolar sockets of the neighboring teeth were filled with cortical and trabecular bones.

**Figure 3:**
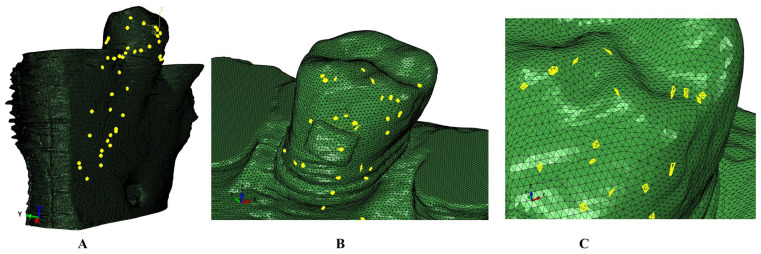
Mesh model of one of the nine 3D models with element warnings: (**A**)—some of the element warnings for the tooth structure; (**B**)—detail of the crown and bracket with element warnings location; (**C**)—detail of the crown with element warnings.

The mesh size had a maximum of 5.06–6.05 million C3D4 tetrahedral elements, 0.97–1.07 million nodes, and a global element size of 0.08–0.116 mm. The mesh surface showed no error and only a few element warnings (e.g., 264 element warnings, approx. 0.0043% for the entire model of 6.05 million C3D4 elements). In Figure 3, one of the models showed 43 element warnings (approx. 0.0062%) for a total number of 690,753 elements of the entire tooth structure: bracket–enamel–dentine–dental pulp–NVB.

The FEA simulations were conducted using ABAQUS 6.13-1 software (Dassault Systèmes Simulia Corp., Maastricht, The Netherlands). Four failure criteria commonly used in dental FEA studies were used: the brittle materials Minimum Principal compressive stress (MinP) and Maximum Principal tensile stress (MaxP), and ductile materials Tresca shear stress (T) and Von Mises overall stress (VM). The applied force was a medium-large 4 N (approx. 407 g/mm) of extrusion, intrusion, rotation, tipping, and translation applied on the bracket (Figure 3). The boundary assumptions were isotropy, linear-elasticity, and homogeneity; perfectly bonded surfaces; and the base of the models encastred (as in most of the dental FEAs).

**Figure 4:**
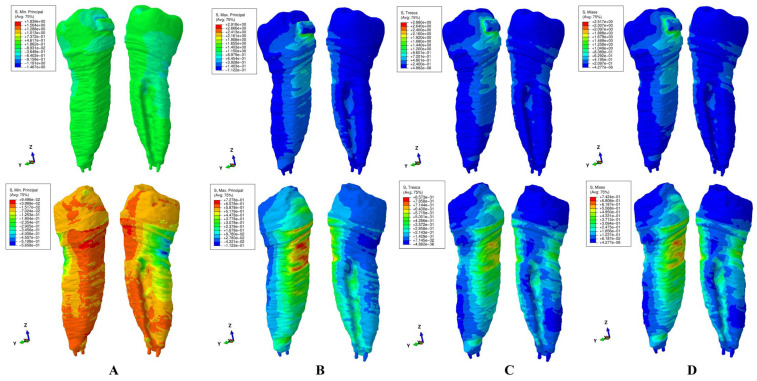
Stress display during extrusion in the tooth (above) and dentine (below) structures using the four failure criteria: (**A**)—Minimum Principal (compressive); (**B**)—Maximum Principal (tensile); (**C**)—Tresca (shear); (**D**)—Von Mises (overall).

**Figure 5:**
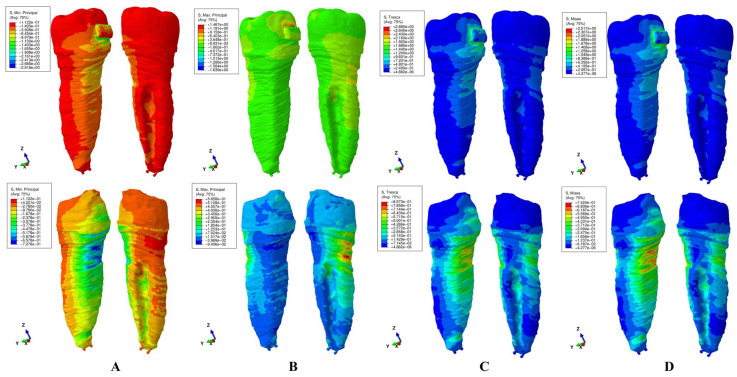
Stress display during intrusion in the tooth (above) and dentine (below) structures using the four failure criteria: (**A**)—Minimum Principal (compressive); (**B**)—Maximum Principal (tensile); (**C**)—Tresca (shear); (**D**)—Von Mises (overall).

**Figure 6:**
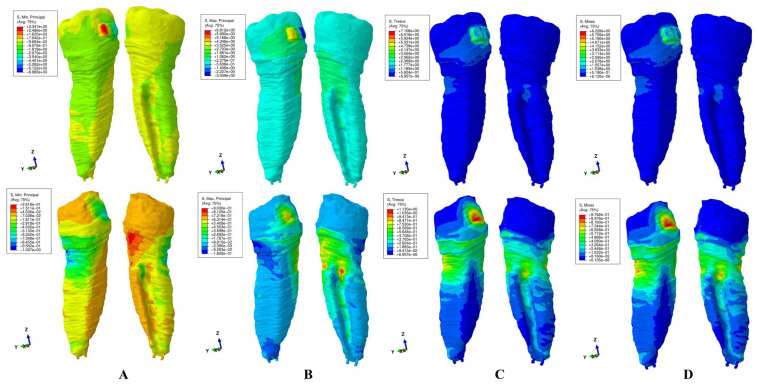
Stress display during rotation in tooth (above) and dentine (below) structures using the four failure criteria: (**A**)—Minimum Principal (compressive); (**B**)—Maximum Principal (tensile); (**C**)—Tresca (shear); (**D**)—Von Mises (overall).

**Figure 7:**
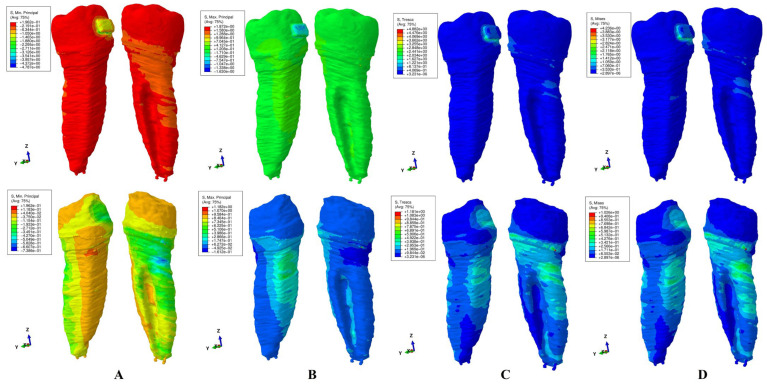
Stress display during tipping in tooth (above) and dentine (below) structures using the four failure criteria: (**A**)—Minimum Principal (compressive); (**B**)—Maximum Principal (tensile); (**C**)—Tresca (shear); (**D**)—Von Mises (overall).

**Figure 8:**
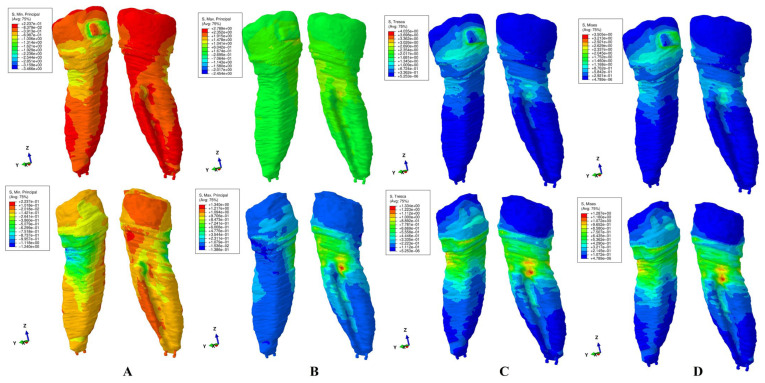
Stress display during translation in tooth (above) and dentine (below) structures using the four failure criteria: (**A**)—Minimum Principal (compressive); (**B**)—Maximum Principal (tensile); (**C**)—Tresca (shear); (**D**)—Von Mises (overall).

The results of these FEA simulations were displayed both as comparative color-coded stress displays (Figure 4, Figure 5, Figure 6, Figure 7 and Figure 8) in tooth structure (bracket–enamel–dentine–pulp–NVB) and dentine structure (dentine–pulp–NVB), and as quantitative results (Table 2). The color-coded stress concentrations with various shades were interpreted as: red-orange (high-intense), yellow-green (medium-average), and blue (low) stresses.

## 3. Results

Our study was performed on nine patients, providing nine intact periodontium 3D models and 180 FEA simulations, with the results displayed in Table 2 and Figure 4, Figure 5, Figure 6, Figure 7 and Figure 8. No differences were seen in gender, age, or periodontal status.

In each of the analyzed structures (entire tooth and dentine–pulp–NVB), the four types of stress were qualitatively and quantitatively displayed, and their biomechanical correctness was assessed.

Quantitatively (Table 2), the average numerical stress values (in KPa) in the tooth structure were higher than those displayed by the dentine–pulp–NVB complex for all five movements and failure criteria. The higher numerical values were seen mostly in the cervical third and around the bracket area. However, a close analysis revealed that when brittle-like material failure criteria were used, some issues related to the amount (higher in the dentine–pulp–NVB structure than in the tooth, e.g., MinP for tipping and rotation) and type of stress (a positive sign instead of negative for MinP in extrusion, rotation, and translation, while for MaxP, a negative sign instead of positive in rotation) were seen (Table 2). No such inconsistencies were seen when the ductile-like material failure criteria were applied. The T numerical values were higher than VM, as expected. Nonetheless, no clear differentiation (brittle- vs. ductile-like) based on only quantitative results could be established.

Qualitatively (Figure 4, Figure 5, Figure 6, Figure 7 and Figure 8), general cervical and coronal color-coded stress was displayed by the tooth structure, without any sign of areas prone to external orthodontic resorption. However, when the dentine structure was analyzed, clear islands of stress concentrations were displayed, suggesting that this area is more prone to the resorptive process. Nevertheless, upon a closer look, various inconsistencies were seen between the brittle- and ductile-like failure criteria during various movements, enabling differentiation among them.

*Extrusion* (Figure 4). Both the Tresca and Von Mises criteria displayed similar stress patterns in both the dentine and tooth. However, while there were no stress differences in the tooth structure (only mostly vestibular cervical stress), in the dentine–pulp–NVB structure, larger vestibular, proximal, and lingual areas of higher red-orange stress were seen in the cervical third for VM when compared with the T criterion. Moreover, the dentine structure displayed extended stress in the entire structure, consistent with known biomechanical behavior. VM seems to show a higher and more extended red-orange stress display than T (which only shows limited orange areas), thus seeming to be closer to the natural clinical behavior.

The brittle-like MaxP (tensile) displayed a similar pattern with T and VM in both the tooth and dentine. The dentine structure displayed mostly vestibular side extended tensile stress (red-orange color-coded stress) and lingual middle and apical third stress. These areas are too extensive from a biomechanical point of view, contradicting clinical knowledge. The MinP displayed almost no compressive stress in the tooth, but showed limited lingual cervical third stress in the dentine structure, with an odd disposition, contradicting known clinical biomechanical behavior. These stress displays seem to be less accurate in representing the known biomechanical behavior.

*Intrusion* (Figure 5). Both T and VM displayed a similar color-coded stress projection as during extrusion. The MinP and MaxP also showed color-coded stress correlations with extrusion movement (MinP-MaxP and MaxP-MinP), due to changes in stress and movement type. The same inconsistencies related to the biomechanical stress display in brittle-like criteria can be seen, contradicting known clinical reports.

*Rotation* (Figure 6). T and VM similarly displayed little amounts of stress in the tooth structure, mostly on and around the bracket. However, when analyzing the dentine structure, the higher red-orange stress is displayed both coronally around the bracket area as well as in the cervical third (distal–lingual and lingual sides). Between the two, the overall stress VM displayed the highest amount of red-orange stress. The compressive MinP displayed limited lingual–distal stress in the cervical third, while the tensile MaxP displayed both lingual and lingual–distal sides with higher stress areas. Both brittle-like criteria displayed a closer high stress resemblance to the ductile-like ones.

*Tipping* (Figure 7). In the tooth structure, both T and VM displayed moderate stress around the bracket area. In the dentine structure, the same moderate stresses, less prone to resorption, were displayed in the lingual third sides. The MaxP tension assessment showed a large extension in the vestibular cervical third of the dentine. The MinP compressive display showed limited high stress on the dentine’s lingual side. T, VM, and MaxP seemed to show less predisposition to external orthodontic resorption, while MinP displayed the contrary.

*Translation* (Figure 8). In the tooth structure, the coronal stress displayed by T and VM seems more extended than the cervical one, but with no sign of higher color-coded stresses. In the dentine structure, both T and VM criteria displayed an identical stress display of red-orange island on the lingual and distal cervical third sides, prone to limited orthodontic external resorption. The MaxP seems not to display any tension in the tooth, and only limited areas of red-orange in the dentine’s lingual cervical third (similarly to T and VM). The MinP compression is displayed in the coronal and cervical third of the tooth structure, with higher compressive stress in the dentine’s distal cervical third. T, VM, and MaxP similarly displayed dentine’s distal–lingual and lingual sides as more prone to orthodontic external resorption, with higher stress concentrations on the lingual side, contrary to MinP, which displayed no lingual stress but showed concentration on the distal–lingual sides.

When both quantitative and qualitative data are correlated, the ductile-like failure criteria seem to maintain accuracy in each of the simulations, while the brittle-like criteria do not seem to be as reliable and accurate. Moreover, according to the simulations, tipping is less prone to developing external orthodontic resorption, while translation displays the highest possibility. About the amount of stress under 4 N of orthodontic force, no significant resorptive risks are seen, except for stress concentrations usually found in the dentine’s cervical third.

## 4. Discussion

Our study is a progressive FEA project focused on determining the proper failure criteria to most accurately describe the clinical biomechanical behavior of dental tissues under orthodontic force and movements [13,14,15,16,17]. Due to the difficulty of conducting this type of study under in vivo conditions, the only viable possibility is to consider finite element studies [2,16]. The advantages of FEA are related to the reduced number of samples used in the study (usually one, but nine in our study) due to limitless possibilities in changing the boundary conditions of the simulations, allowing an unlimited number of experiments [1,2,15,16,26,27,28]. The limits of the study are mainly related to the fact that a simulation cannot identically reproduce clinical tissue biomechanical behavior [2,16,19]. However, the engineering field has set up mandatory conditions to be used in FEA simulations to provide accurate results, and the fact that FEA is widely used worldwide in the development of various industries proves that its results can be accurate and reliable. In the dental field, these mandatory conditions are related to the anatomical accuracy of the 3D models, reliable boundary conditions, and proper failure criteria [15]. However, dental numerical studies rarely follow the above-mentioned approach, leading to less accurate results [13,14,15,16,17]. By carefully analyzing these issues, improvements can be achieved in both research and clinical fields.

Our results showed that when analyzing the entire tooth structure during all five movements and independently of the failure criteria (ductile- or brittle-like), the color-coded stress display seems not to be relevant in pinpointing areas of orthodontic external resorption. These results are in contradiction with previous older reports [8,20,21,22,24,25,36,37], but in line with a newer one [13]. Based on these simulations, 4 N of orthodontic force does not seem to be prone to induce enough stress concentration to induce orthodontic external root resorption due to the reported absorption–dissipation tissular stress ability [13,14]. However, when analyzing the dentine structure, limited islands of color-coded higher stress concentrations were displayed around the cervical third of the structure, surrounding the same location independently of the failure criterion used, in line with our previous reports [13,14] and others [9]. This seems to support previous reports [13,14,15,16,17] about the absorption–dissipation role of all dental tissue components.

Higher stress areas (red-orange) were more prone to suffering from external orthodontic resorption than the middle (yellow-green) and lower (blue) stress areas, in line with [13,14].

All four failure criteria seem to similarly display a higher stress area, independently of the type of stress they analyze; however, the ductile-like Tresca and Von Mises seem to display the color-coded stress concentrations more accurately than the brittle-like MaxP and MinP. This is also supported by the quantitative results, with various inconsistencies that are difficult to biomechanically explain, and is related to the fact that all dental components biomechanically behave more like ductile-like material than brittle-like [13,14,15,16,17], despite their chemical inorganic composition, as supported by current research flow data.

From a physical engineering and biomechanical point of view, 4 N is considered an extremely low amount of force [16,17]; thus, there seems to be no difference between biomechanical tissue behavior under less than 1 N and more than 1 N, in line with previous reports [13,14,15,16,17].

In intact periodontium and under 4 N orthodontic force, the cervical third area is more prone to suffer from external orthodontic resorption induced by orthodontic movement (confirmed by all four failure criteria stress displays), as reported [13,14,23], but correlated with the time of appliance, as reported by the clinical data [3,5,6,10,11,12]. The coronal dentine under the bracket position also seems to be more prone to a high amount of stress, which might also lead to resorption islands, as reported [13,14].

Regarding the difference between Tresca and Von Mises employment, both are used to predict the onset of permanent deformation under a complex state of stress [16]. Von Mises is considered to be generally more accurate from the engineering point of view when predicting the onset of yielding (Figure 4D, Figure 5D, Figure 6D, Figure 7D and Figure 8D, more red and less orange) [16]. Tresca is more conservative and, from the engineering point of view, predicts yielding of the material at a lower stress level (as displayed in Figure 4C, Figure 5C, Figure 6C, Figure 7C and Figure 8C, less red and more orange); therefore, it is considered “safer” and a more conservative choice if the aim is to ensure that no yielding occurs (when plotted the Tresca forms a hexagon contained by the Von Mises ellipse) [16]. Since external orthodontic resorption is difficult to anticipate and predict [13,14] and is a totally undesirable clinical phenomenon, the use of the Tresca failure criterion is more reliable than Von Mises [16].

The boundary assumptions could influence the numerical simulation’s accuracy [13,14,15,16,17]. Thus, an aspect that might be of importance when selecting the failure criteria is the fact that, from the engineering point of view, Tresca is appropriate for anisotropic materials, while Von Mises is appropriate for isotropic ones [13,14,15,16,17]. The dental tissues are considered to be anisotropic materials [15]. Another issue is related to the linear elasticity biomechanical behavior of dental tissues under small loads [16]. Again, from the engineering point of view, under small/extremely small loads, all materials follow the linear-elasticity assumption, with 4 N being covered by this aspect [16,17]. Moreover, previous reports of our team proved the constant stress display for 0.6 and 1.2 N for the Tresca failure criterion [13,14]. Thus, multiple FEA studies [3,8,28,29,30] employed isotropy, linear-elasticity, and perfectly bonded interfaces as proper boundary assumptions (as herein) for FEA.

When employing the brittle-like failure criteria, the entire structure displays both tension and compression variations. Thus, those seem to be biomechanically less relevant than the overall and/or shear stress when assessing orthodontic external resorption, making the ductile-like Tresca and Von Mises more accurate and better suited for this type of FEA [17].

When analyzing the orthodontic external resorption, FEA studies need to focus more on the dentine component to assess the stress concentration areas.

Our results reported that under 4 N (approx. 400 g/mm) rotation, translation, intrusion, and extrusion movements seem to be prone to various levels of external orthodontic resorption, in line with previous reports [23], while tipping movement seems likely to be safer. The reported apical third resorption [23] was not confirmed by our results and contradicted by other results [13,14]. Nevertheless, the rotation and extrusion type of resorption were in line with [13,14,23]. A lower amount of stress was seen in the apical third of the root, in line with a clinical report [5], but contradicting another one [4].

Multiple new FEA studies using the Von Mises criterion (abfraction cavities simulation under 300 N of oblique force simulating masticatory forces over endodontically treated lower first molars [3,29], premolars [28], and incisors [32]) reported both extended vestibular cervical stress at the abfraction site as well as at the applied force contact point with the enamel. However, some of these color-coded displays could raise concerns about clinical accuracy due to their extreme extensions, biomechanically suggesting an imminent failure of the tooth.

A new numerical report [29] assessing the endodontically treated lower molars displayed extended cervical third stress in the entire tooth structure, but with a different display from our result. Another report assessing the cervical resorption in restored incisors displayed extended red areas in the entire palatal side of the root [32], suggesting extended areas of resorption, which is biomechanically and clinically incorrect. Other reports [3,28] analyzed the entire tooth structure and displayed extended areas of color-coded red stress concentrations, both in the enamel and cervical third area, of extreme extension and uniform in color-coded red (for 300 N), compared with our models with visible limited color-coded yellow-green stress concentrations (under 4 N) in both the enamel and cervical third, which are closer to clinical reality. The high red stress concentration island, prone to resorption areas, appeared only when analyzing the dentine structure, signaling the limited potential for orthodontically induced root resorption. Another issue is related to the extremely high amount of VM-reported stress by the above-mentioned new numerical studies: 100–258 GPa [38] (for 300 N, intact–damaged tooth), 95–152 MPa (for 100 N, damaged tooth) [32], 20–30 MPa (intact tooth) [29], 33–50 MPa (intact tooth) to 95–100 MPa (abfraction damaged) [28], and 27–38 MPa (intact tooth) to 116 MPa (abfraction damaged) [3] (for 300 N) vs. 0.84 MPa (for 4 N of intrusion over intact tooth, VM, Table 2) localized red-orange islands herein. These are probably due to differences in the 3D models’ anatomical accuracy and interior design (e.g., fix the mesh size of 0.5 mm) [3,28,29] vs. a variable 0.08–0.116 mm herein, automated [3,28,29] vs. manual segmentation of the models, amount, and direction of force. Another issue that might influence the color-coded stress distribution is the fact that all previous studies simulated various depths and forms of abfraction/cavities of cervical root resorption [3,28,29,32,34,35,38,39,40] and intact tissue, as herein. To the best of our knowledge, this study is the first to approach this failure criteria analysis regarding the orthodontic resorption (except for our previous [13,14]). No motivation for selecting the Von Mises failure criterion was found in previous numerical analyses [3,28,29,31].

Only one recent study [33] about the orthodontic external apical root resorption reported results closer to our study. Thus, 0.6 N of translation had been reported to induce the highest stress numerical values among the four simulated movements (rotation has not been simulated) over an upper central incisor. In our study, we found that the rotation induced the highest quantitative stress, closely followed by translation, which is in line with Rodrigues Fonseca Tavares et al.’s [33] report. Since both studies showed similarities in method, the correlations are useful. The reported quantitative cervical stress for intact tooth and periodontium for 0.6 N was 250 KPa (intrusion), 257 KPa (extrusion), 55 KPa (tipping), and 260 KPa (translation) [33] vs. that for 4 N, which was 838 KPa (intrusion), 838 KPa (extrusion), 707 KPa (tipping), and 1461 KPa (translation). When assessing the differences between the two applied forces (0.6 N vs. 4 N), our amount of stress is lower, with an average of about 18–50%, except for tipping, which is about 50% higher. These quantitative numerical differences are due to the type of tooth and topography (upper central incisor vs. lower second premolar here). The qualitative results (color-coded stress display) for the VM criterion showed close resemblance related to the areas of higher stress, as well as the red-orange stress for all movements. Nevertheless, in our study, the high red-orange concentrations prone to resorption islands are seen only when examining the dentin-pulp-NVB component, while the other report [33] displayed it for the entire tooth structure. These qualitative differences are probably due to the boundary assumption since both Young’s modulus and Poisson’s ratio coefficient (mechanical properties) are closely related. The mesh size of 0.5 mm and number of nodes/elements (e.g., 536,441/319,731) [33] vs. 0.08–0.116 mm, 5.06–6.05 million C3D4 tetrahedral elements, 0.97–1.07 million nodes herein might also make a difference (anatomical accuracy). Another study [13] of our team (0.6 N and 1.2 N, Tresca shear stress, intact periodontium, five movements) reported similar qualitative and quantitative results.

Under 4 N of force, all quantitative numerical results exceeded the maximum amount of hydrostatic pressure (MHP) of 4.7–16 KPa [8,13,14,15,16,17], signaling that the red-orange color-coded stress concentration area islands, almost 45–85 times higher than MHP, could induce localized islands of orthodontic external root resorptions. Nevertheless, even 4 N of force is not mandatory to induce orthodontic resorption, since the optimal amount of force is still a subject of controversy [8,13,14,15,16,17]. However, ever since, the resorptive process has been highly influenced by the amount of force, time, anatomical topography, and other local particularities [6,11,13,14,15,16,17]; therefore, knowing the areas more prone to suffer from resorption (FEA being the only known method [13,14,15,16,17,28,29,30,31,41]) is of importance in planning clinical orthodontic treatment.

A single study [8] was found to actually describe the islands of orthodontic root resorption in line with our results. This FEA simulation associated with a clinical study involved ten first lower premolars under a tipping force of 0.245 and 2.2 N for 12 weeks, reporting localized islands of external root resorption in the entire root surface, but with no identical pattern other than the side of the root (lingual and vestibular, as herein) [8]. Our results partially agree with these reports; nevertheless, our models displayed a more localized area (cervical third) and limited and low-depth areas. The FEA simulation [8], however, exceeded 85% of MHP, but using the hydrostatic pressure failure criteria (with limited accuracy in dentistry [24,25]), since its design is for liquids and not ductile/brittle-like materials, and does not take into consideration the interior shear stress [13,14,15,16,17]. The color-coded stress results did not match the clinical resorption areas (probably influenced by other local clinical factors), thus analyzing less anatomically accurate 3D models (e.g., element size of 1 mm, 204,369 elements, for a mandibular model including six teeth). It must be acknowledged that anatomical correctness is mandatory for accurate results (many dental studies reported inconsistencies [24,25,36,37]). Despite the mandatory sample size of one required by the numerical analyses, to better follow the stress patterns during the biomechanical behavior, the selected number of models was greater than the minimum required. The mesh convergence testing was also performed.

The amount of optimum orthodontic force in intact periodontium is still a subject of debate [13,14,15,16,17], with various reports ranging from 0.2 to 4 N. Since our previous team studies investigated light and medium forces, and the amount of force impacts the boundary conditions that influence biomechanical behavioral description, a greater force of 4 N is still usable in clinical conditions.

Our study agrees with some reports [8,18,25] regarding the premise of individually unpredictable resorptive reactions [6,7,8,10,11,12,18,36,41] induced by variable time-dependent higher points of pressure [23]. The above-mentioned are dependent on patient reactivity, anatomical variations, and biomechanical particularities, suggesting the need for separate assessment of each tooth, which is also in line with our study.

## 5. Conclusions

In intact periodontium, under 4 N and five orthodontic movements, tipping seems less prone to external orthodontic resorption than translation, extrusion, intrusion, and rotation, which displayed areas of high stress concentrations in the cervical third of the root.

Higher stress concentrations were clearly displayed only when the dentine–pulp–NVB components were analyzed separately, not when the entire tooth structure was assessed.

An applied orthodontic force of 4 N can induce limited islands of external orthodontic root resorption (intrusion–extrusion on the vestibular side, rotation–translation on the lingual and distal–lingual sides).

The ductile-like failure criteria maintained the accuracy of the results across all FEA simulations, whereas the brittle-like criteria exhibited various inconsistencies, both quantitative and qualitative.

## 6. Clinical Implications

Despite its clinical relevance, only a few recent studies on external orthodontic-induced resorption have been published. These numerical analyses (the only suitable study method) identified several biomechanical inconsistencies when compared with known clinical behavior. From a research perspective, the study allows for reviewing the differences caused by using the most common failure criteria, helping determine which is the most appropriate and thereby advancing clinical and theoretical knowledge. Additionally, by evaluating the results and comparing them with other reports, the methodological issues influencing result accuracy are recognized, leading to a method and failure criteria that ensure precise outcomes. Clinically, the study highlights stress concentration areas induced by orthodontic movements that are more susceptible to resorption. It also demonstrates that a force of 4 N is not necessary to induce resorption, offering new insights into optimal applied forces. Furthermore, it shows that rotation, closely followed by translation, is more prone to the resorptive process, while tipping is the least susceptible. Consequently, both clinicians and researchers gain a better understanding of tissue biomechanical behavior and how numerical studies can provide accurate results.

## Figures and Tables

**Table 1:** Physical properties of materials.

Materials	Young’s Modulus, E (GPa)	Poisson Ratio, ʋ	Refs.
Enamel	80	0.33	[13,14,15,16,17]
Dentin/Cementum	18.6	0.31	[13,14,15,16,17]
Pulp and NVB	0.0021	0.45	[13,14,15,16,17]
PDL	0.0667	0.49	[13,14,15,16,17]
Cortical bone	14.5	0.323	[13,14,15,16,17]
Trabecular bone	1.37	0.3	[13,14,15,16,17]
Stainless-steel Bracket (Cr-Co)	218	0.33	[13,14,15,16,17]

**Table 2:** Quantitative amounts of stress (KPa) produced by 4 N.

Movement	Failure Criteria	Component	Apical	Middle	Cervical	Coronal
**extrusion**	**MinP**	S	−365.10	−365.10	−641.00	−1193.00
		D	−126.10	−126.10	−565.90	**39.91**
	**MaxP**	S	393.10	647.20	898.20	1152.00
		D	378.00	518.30	728.10	448.00
	**T**	S	490.02	490.02	729.33	967.90
		D	288.50	288.50	716.60	359.30
	**VM**	S	419.60	419.60	838.10	840.10
		D	245.23	245.23	682.60	310.40
**intrusion**	**MinP**	S	−393.80	−647.40	−880.10	−1151.00
		D	−379.20	−519.80	−728.80	−379.80
	**MaxP**	S	365.20	365.20	641.30	641.30
		D	348.60	348.60	567.80	127.30
	**T**	S	490.02	490.02	729.33	967.90
		D	288.50	288.50	716.60	359.30
	**VM**	S	419.60	419.60	838.00	840.10
		D	245.23	245.23	682.60	310.40
**rotation**	**MinP**	S	765.30	765.30	**765.30**	765.30
		D	−182.30	−182.30	−1068.10	−735.10
	**MaxP**	S	−584.80	1062.10	1062.10	2704.00
		D	179.30	270.10	904.00	904.00
	**T**	S	593.20	593.30	1194.55	2377.23
		D	472.10	472.10	1131.10	1131.10
	**VM**	S	519.10	519.10	1039.30	2077.20
		D	409.10	409.10	980.20	980.20
**tipping**	**MinP**	S	−635.40	−635.40	**−635.40**	−635.40
		D	−428.10	−428.10	−739.10	−428.10
	**MaxP**	S	413.00	413.00	413.00	413.00
		D	175.70	287.60	287.60	62.80
	**T**	S	407.00	407.00	814.20	1222.10
		D	394.10	394.10	788.10	394.10
	**VM**	S	354.20	354.20	707.20	1060.10
		D	343.10	343.10	685.10	343.10
**translation**	**MinP**	S	223.50	−392.30	−1315.00	−1007.60
		D	−143.10	−143.10	−1242.10	−143.10
	**MaxP**	S	605.20	1040.00	1916.00	1916.00
		D	108.90	725.30	1341.10	232.10
	**T**	S	337.1	672.5	1348.1	1348.1
		D	333.6	445.6	1335.1	445.5
	**VM**	S	293.20	584.20	1461.00	1753.00
		D	214.60	322.60	1181.00	429.10

S—tooth structure: enamel, dentine, dental pulp, neuro-vascular bundle, stainless-steel bracket. D—dentine structure: dentine, dental pulp, neuro-vascular bundle, Bold and gray color background—numerical inconsistencies.

## Data Availability

The original contributions presented in this study are included in the article. Further inquiries can be directed to the corresponding authors.
